# Supplementary figures and images for: Phylogeography of the Mesa Silverside fish Chirostoma jordani (Woolman, 1894) throughout the Mexican Plateau
Source: PeerJ. 2024 Dec 12;12:e18256. doi: 10.7717/peerj.18256 (PMC11646419; doi:10.7717/peerj.18256)

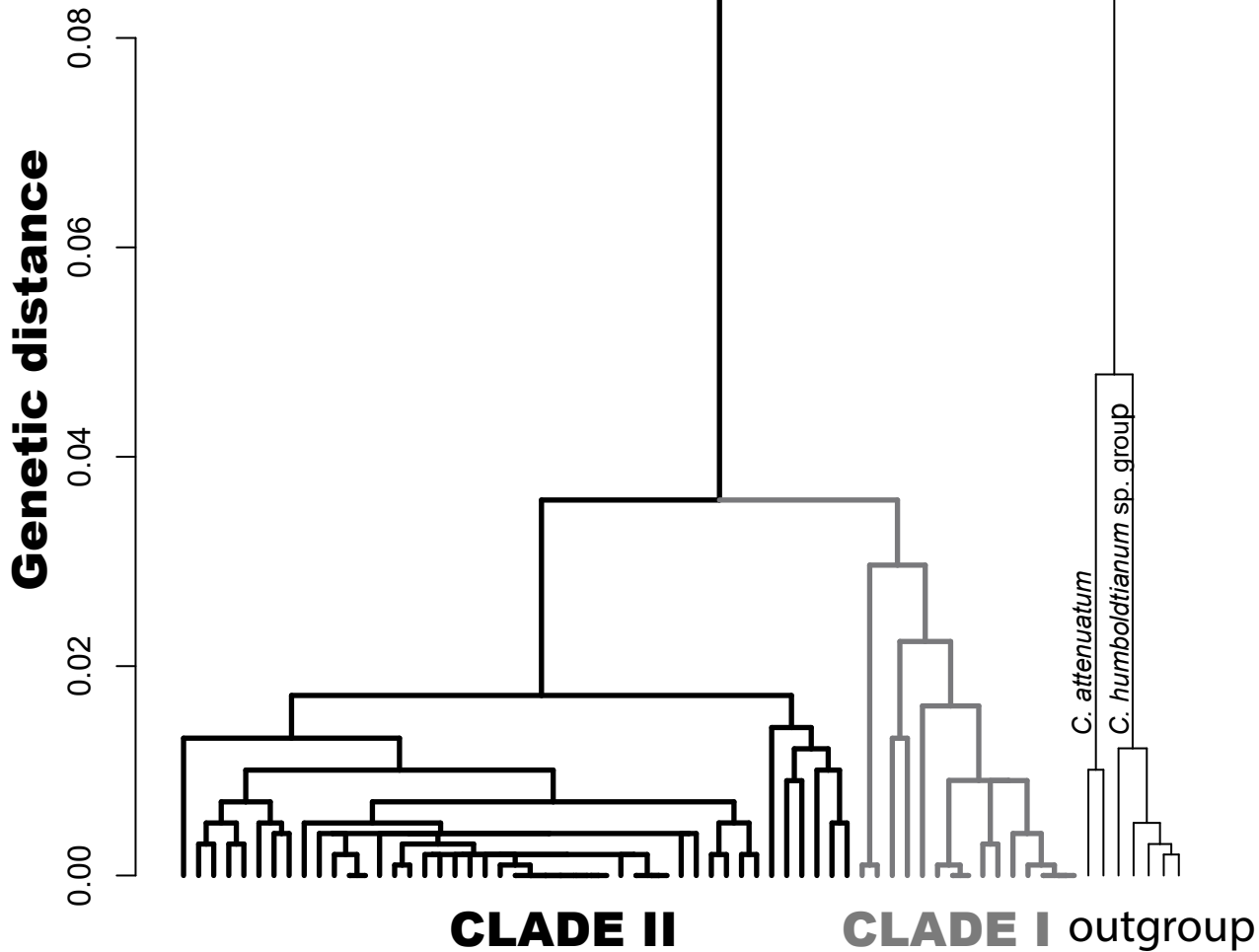

Supplement: Supplemental Information 2 — y label = uncorrected genetic distance, x label = individuals clusters acording to main clades recovered in gene trees. Grey branches represented individuals recovered in the main clade I, and black branches represented idividuals into the main clade II. The cluster was acording to results foun in gene trees. [file peerj-12-18256-s002.pdf]

# Genetic distance

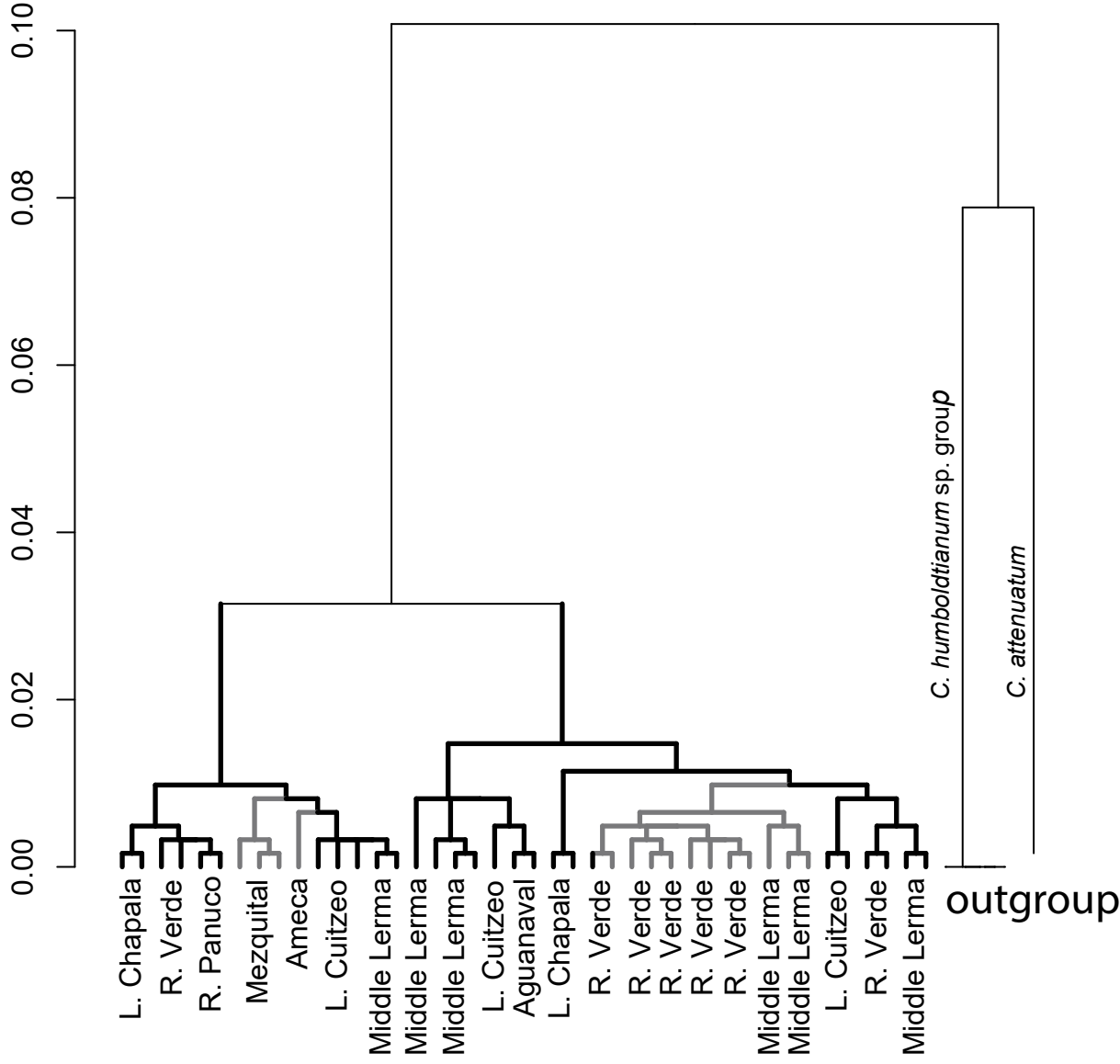

Supplement: Supplemental Information 3 — y label = uncorrected genetic distance, x label = individuals clusters acording to main clades recovered in gene trees. Grey branches represented individuals recovered in the main clade I, and black branches represented idividuals into the main clade II. The cluster was acording to results foun in gene trees. [file peerj-12-18256-s003.pdf]
