# Supplementary material for: Phylogeography of the Mesa Silverside fish Chirostoma jordani (Woolman, 1894) throughout the Mexican Plateau
Source: PeerJ. 2024 Dec 12;12:e18256. doi: 10.7717/peerj.18256 (PMC11646419; doi:10.7717/peerj.18256)
Supplement: Supplemental Information 4 — Amplification process was conducted in a reaction of 25 µL, containing 50-100ng DNA, 2.5 mM of 1X buffer, 1.5 mM MgCl2, 2.5 µM dNTP mix (µM 10), 10 pmol of each primer, 1 unit of Taq DNA polymerase (Invitrogen), and distilled water to bring the reaction volume to 25 µL. [file peerj-12-18256-s004.pdf]

Phylogeography of the Mesa Silverside fish *Chirostoma jordani* Woolman, 1894 along the Mexican Plateau.

Isaí Betancourt-Resendes, Rodolfo Pérez-Rodríguez, Kyle R. Piller, and Omar Domínguez-Domínguez

Supplementary Table 1. PCR conditions and best-fit model for each marker used.

Amplification process was conducted in a reaction of 25 µL, containing 50-100ng DNA, 2.5 mM of 1X buffer, 1.5 mM $MgCl_2$, 2.5 µM dNTP mix (µM 10), 10 pmol of each primer, 1 unit of Taq DNA polymerase (Invitrogen), and distilled water to bring the reaction volume to 25 µL.

Locus	Primers	Denaturing	Cycles	Denaturing	Annealing	Extension	Final extension	Best-fit model
Cytochrome b (*cytb*)	Glud-G & H16460	94°C-2min	35	94°C-45s	48°C-1min	72°C-1min	72°C-5 min	TIM2+I
hypervariable control region (*dloop*)	RCA & RCE	94°C-3min	35	94°C-30s	54-56°C-45s	72°C-1min	72°C-5 min	TIM2+G
first intron of ribosomal protein S7 (*S7*)	S7RPEX1F & S7RPEX1R	94°C-1min	35	94°C-30s	56-58°C-45s	72°C-45s	72°C-5 min	GTR+G

Primers sequence

Cytochrome b

Glud-G: 5'-TGACTTGAARAACCAYCGTTG-3'

H16460: 5'-CGAYCTTCGGATTAACAAGACCG-3'

Hypervariable D-loop region

RCA: 5'-TTCCACCTCTAACTCCCAAAGCTAG-3'

RCE: 5'-CCTGAAGTAGGAACCAGATG3'

First intron of Ribosomal Protein S7

S7RPEX1F: 5'-TGCCCTCTTCCTTGGCCGTC3'

S7RPEX1R: 5’-AACTCGTCTCGCTTTTCGCC-3’
