# Supplementary material for: Phylogeography of the Mesa Silverside fish Chirostoma jordani (Woolman, 1894) throughout the Mexican Plateau
Source: PeerJ. 2024 Dec 12;12:e18256. doi: 10.7717/peerj.18256 (PMC11646419; doi:10.7717/peerj.18256)
Supplement: Supplemental Information 5 [file peerj-12-18256-s005.pdf]

Phylogeography of the Mesa Silverside fish *Chirostoma jordani* Woolman, 1894 along the Mexican Plateau. Isaí Betancourt-Resendes, Rodolfo Pérez-Rodríguez, Kyle R. Piller, and Omar Domínguez-Domínguez

Supplemantary Table 2. Genbank accesion number of ingroup samples for all loci.

Locality	Biogeographic region/Coordinates (UTM)/altitude(msnm)	*Cytochrome b* GenBank Accesion	*Hipervariable dloop* GenBank Accesion	Firs intron of Ribosomal Protein *S7* GenBank Accesion
La Vega	Ameca/ 619800.499-2287120.82 N-W/1258	PP475811-PP475814	PP475940-PP475944	PP484970
El Tesorero	Bolaños-Santiago/709002.563-2526197.09 N-W/2121	PP475815-PP475816	PP475945-PP475947	PP484946-PP484947
Tejocotal	Cazones/589749.097-2226765.78 N-W/2127	PP475817-PP475821	PP475948-PP475956	PP485010-PP485015
Cajititlan	Santiago/ 674261.47-2258395.41 N-W/1554	PP475822-PP475824	PP475957-PP475961	PP484948-PP484952
Petatan	Chapala/ 722833.606-2230914.75 N-W/1523	PP475825-PP475828	PP475962-PP475968	PP485004-PP485009
Los Negritos	Chapala/ 750047.08-2220053.79 N-W/1520	PP475829-PP475841	PP475969-PP475973	nd
San Juanico	Cotija/ 741703.912-2196193.06 N-W/1839	PP475842-PP475846	PP475974-PP475978	PP484965-PP484969
Andocutiin	Cuitzeo/ 1305189.933-2206884.41 N-W/1835	PP475847-PP475852	PP475979-PP475983	PP484975-PP484979
Balneario Huingo	Cuitzeo/ 308083.243-2202811.12 N-W/1842	PP475853-PP475856	PP475984-PP475988	PP484981-PP484982
Sengio	Cuitzeo/347332.761-2186273.65 N-W/2383	nd	PP475989-PP475992	PP484980
Orandino	Lower Lerma/ 779796.889-2208878.84 N-W/1570	PP475857-PP475862	PP475993-PP476000	PP485016-PP485022
Presa de Garabato	Santiago/ 739804.272-2282737.64 N-W/1714	PP475863-PP475864	PP476001-PP476002	nd
Magdalena	Magdalena/ 705892.034-2312789.4 N-W/1364	PP475865-PP475868	PP476003-PP476009	PP484971-PP484974
Gpe Aguilera	Mezquital/528970.567-2704378.12 N-W/1996	PP454718-PP454722	PP454723-PP454730	PP454731-PP454734
Presa Angamacutiro	Middle Lerma/ 215027.129-2228144.01 N-W/1702	PP475869-PP475874	PP476010-PP476012	nd
Arroyo Neutla	Middle Lerma/ 306311.287-2289428.77 N-W/1885	PP475875	PP476013	PP484953
Echevereste	Middle Lerma/ 224023.558-2344597.77 N-W/1897	PP475876-PP475885	PP476014-PP476023	PP484990-PP484995
Taretan	Middle Lerma/ 257244.148-2298541.97 N-W/1746	PP475886	PP476024-PP476025	PP484989
San Francisco del Rincon	Middle Lerma/ 204225.233-2331772.92 N-W/1781	nd	PP476026	nd
Yuriria	Middle Lerma/	PP475887-	PP476027-	PP484988

	280552.445-2240911.7 N-W/1739	PP475889	PP476032	
Lago de Guapango	Panuco/ 425748.229-2208809.93 N-W/2621	PP475890-PP475892	PP476033-PP476036	nd
Tepeji del Rio	Panuco/ 467199.404-2207128.25 "N-W/2119	PP475893-PP475895	PP476037-PP476040	PP484943-PP484944
Belem del Refugio	Verde-Santiago/ 765619.211-2383092.54 N-W/1720	PP475896	PP476041	PP484945
Lagos de Moreno	Verde-Santiago/ 191450.178-2363469.41 N-W/1854	PP475897-PP475902	PP476042-PP476047	PP484996-PP485003
Nochistlan	Verde-Santiago/ 734535.703-2369224.73 N-W/1904	PP475903-PP475908	PP476048-PP476053	PP484959-PP484961
La Paz Dam	Verde-Santiago/ 230865.787-2418623.33 N-W/	PP475909-PP475910	PP476054-PP476055	nd
Chichimeco Dam	Verde-Santiago/ 771310.885-2436011.25 N-W/1953	PP475911-PP475914	PP476056-PP476063	PP484983-PP484987
Ojuelos	Verde-Santiago/ 206880.187-2409825.03 N-W/2077	PP475915-PP475919	PP476064-PP476069	PP485032-PP485034
Rio Chilerillo	Verde-Santiago/ 7637116.407-2400295.99 N-W/1766	PP475920-PP475923	PP476070-PP476078	PP485023-PP485031
Rio Verde-Balneario Las flores	Verde-Santiago/ 726741.722-2324472.96 N-W/1504	PP475924-PP475928	PP476079-PP476087	PP484954-PP484958
Rio Verde-Sn Nicolas de las Flores	Verde-Santiago/ 754349.293-2357029.14 N-W/1668	PP475929-PP475932	PP476088-PP476093	PP484962-PP484964
San Isidro	Verde-Santiago/794547.619-2323207.65 N-W/2091	nd	nd	nd
Cuemanco	Mexico Valley/ 490185.98-2134495.23 "N-W/2238	PP475933-PP475939	PP476094-PP476095	nd

nd= sequences were not submited to genebank

Supplemantary Table 2. GenBank accession number of outgroup samples for all loci.

Specie	Locality	Biogeographic region/ Coordinates (GPS)/ altitude(msnm)	*Cytochrome b* GenBank Accession	*Hipervariable dloop* GenBank Accession	Firs intron of Ribosomal Protein *S7* GenBank Accession
Chirostoma estor	Patzcuar o Lake	Patzcuaro/ 19.4395 N - 101.7264 W/ 2030	PQ240566	PQ240571	MG747647
	Zirahuen Lake	Zirahuen/ 19.4395 N - 101.7264 W/2080	PQ240568	PQ240573	PQ240577
Chirostoma humboldtianum	Zacapu Lake	Middle Lerma/ 19.8241 N - 101.6405 W/1980	PQ240567	PQ240572	PQ240576
Chirostoma chapalae	Chapla Lake	Chapala/ 20.1626 N - 102.8679 W/1530	PQ240569	PQ240574	PQ240578
Chirostoma sphyraena	Chapla Lake	Chapala/ 20.1626 N - 102.8679 W/1530	PQ240570	PQ240575	PQ240579
Chirostoma attenuatum	Patzcuar o Lake	Patzcuaro/ 19.4395 N - 101.7264 W/2030	MG592216	nd	MG98486
	Zirahuen Lake	Zirahuen/ 19.4395 N - 101.7264 W/ 2080	MG592228	nd	MG598463

nd= sequences were not submited to genebank
